# NHS England invites 75–80-year-olds for their first respiratory syncytial virus (RSV) vaccines – why should not those in their 80s be included too?

**DOI:** 10.1080/20523211.2025.2477761

**Published:** 2025-03-19

**Authors:** Hamid A. Merchant

**Affiliations:** Department of Bioscience, School of Health, Sport and Bioscience, University of East London, London, UK

Respiratory syncytial virus (RSV), known for its infant and child mortality, can cause severe lung infections in older adults that can lead to life-threatening pneumonia. It has been estimated that RSV accounts for 175,000 GP visits annually for 65 years and over in the UK (Fleming et al., 2015) and is responsible for up to 7500 deaths in older adults every year (Hardelid et al., 2012).

RSV vaccine has been long anticipated to reduce the RSV disease burden and associated winter pressures on NHS. Following the recent approval of the RSV vaccine by MHRA and other regulatory authorities last year, NHS England invited over 1.3 million older adults aged 75–80 years earlier in February 2025 to take a free RSV vaccine (NHS, 2025). However, people over 80 years of age were not included in this campaign and refused a free RSV jab on NHS upon request. Over 80s were referred to take the RSV jab in private which is priced very high at £245 per single dose available at community pharmacies, unlike flu jabs which are usually priced under £15 at a local pharmacy in private (Boots, 2024). This has caused anxiety among the elderly population and there have been several media reports of frustration in people who felt ignored by being left out from the NHS’s first phase of RSV immunisation (Saga, 2024).

The pivotal vaccine efficacy trials for RSV unfortunately under-represented individuals over 80s leading to a lack of efficacy data in this age group. It can be argued that there were still a large number of over 80 years participants in the trial, but among those over 80 years who participated in the trial, not many caught the virus during the trial duration making it difficult to accurately calculate vaccine efficacy in this age group. Joint Committee on Vaccination and Immunisation in the UK (JCVI) in their meeting in October 2024, therefore, were not confident about the protection that the vaccine will offer in this age group (JCVI, 2024).

For older subjects, generally, it is difficult to participate in such trials due to stringent eligibility criteria, for instance, the presence of comorbidities or compromised immune system. The JCVI statement had said ‘advice for the programme would be guided by emerging evidence on duration of protection and disease incidence’ and that an extension to the initial programme would be considered when there was more certainty about protection in the very elderly and the real-world impact of the programme in the 75- to 80-year-old category. The Committee agreed that it would need to formally review in detail the evidence for a potential extension to the programme for the very elderly and under 75 years of high-risk groups (JCVI, 2024).

Apart from the vaccine efficacy data, the decision from JCVI was informed by the modelling of the impact and cost-effectiveness of the immunisation strategy, suggesting immunisation in 75+ was more cost-effective than 65+; however, JCVI acknowledged that the burden of RSV in older adults was less well understood and underestimated by existing surveillance.

The vaccine trials were originally designed to assess the vaccine efficacy in elderly aged 60 years or over (GSK, 2024; Pfizer, 2024; Walsh et al., 2023), and as such, were not designed to age stratify to this degree to isolate individuals over 80 years old. The vaccines were also approved by the regulatory authorities, for instance, MHRA in the UK, and were licensed for use in individuals aged 60 and over (UKHSA, 2025a), and did not discriminate those over 80 years of old.

**Figure 1.**
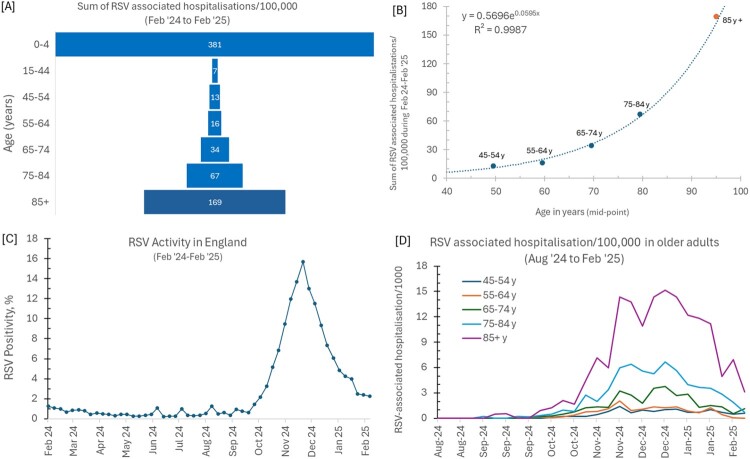
[A] Sum of RSV-associated hospitalisations/100,000 people by age from Feb ‘24 to Feb ‘25, [B] Exponential increase in RSV-associated hospitalisations in older adults with age; for 85y+, mid-point was estimated from exponential fit, [C] RSV positivity of people receiving a PCR test in England from Feb ‘24 to Feb ‘25. A typical RSV season in England starts in October, peaks in December and declines by March. Figure plotted using data from UKHSA RSV dashboard (UKHSA, 2025b).

JCVI originally advised an RSV one-off immunisation campaign for 75 years old and above with a strategy covering several age cohorts to assess its impact with its future delivery and implementation to be determined through further consultation between NHS England, DHSC, UKHSA and the devolved administrations. However, recommended to offer this to only 75–80-year-olds during the first phase to make it similar to the shingles immunisation programme (JCVI, 2024). It is not clear as to why the RSV rollout must follow Shingles, it may rather follow the risk-based approach that was used during the COVID-19 vaccine rollout and the RSV vaccine should have been offered to all over 80s who are most vulnerable to RSV associated hospitalisation among older adults (Figure 1). The limited evidence in pivotal COVID-19 vaccine trials was not a barrier to vaccine rollout in older adults who were at the most risk of adverse outcomes from Covid. Interestingly for Shingles, the new modelling data showed a ‘clear cost-effective benefit’ for preventing shingles and associated post-herpetic neuralgia resulting in a recommendation in Nov 2024 to extend the immunisation programme to over 80s (DHSC, 2024).

The vaccine efficacy data, on the other hand, do not give evidence either that the vaccine is ineffective in individuals over 80s. The strict age cut-off at 80 years is unfortunate and may have ethical implications for not offering protection to those who are most vulnerable to adverse outcomes from the RSV. Offering vaccines to under-80’s for free in NHS and asking over-80s to wait or take it in private at significant costs is against the ethos of NHS. The UKHSA RSV surveillance data suggests largest number of RSV-associated hospitalisations occurred in those 85 years old and over, the highest after children under 4 years who remain most vulnerable to the virus (Figure 1). There is an exponential increase in hospitalisation rate in older adults when the age increases over 80s, making this age group most vulnerable to the virus.

It is, therefore, suggested that NHS should reconsider their decision and offer RSV vaccine to those over 80s in the first phase. This can be offered on an elective basis for this cycle for all over 80s who do not wish to wait any further. Since vaccine stocks may not be fully utilised due to anticipated poor uptake from the eligible populations, it will be best to make this available to those over 80s who are keen to take the vaccine and are likely to benefit the most.

A typical RSV season in the UK starts in October, peaks in December and declines by March (Figure 1C). We have already missed the peak for this season and if the current immunisation campaign were to protect from the virus for the next season (as the new evidence suggests), it is only wise to include those over 80s in the NHS immunisation campaign in the current phase. This will also provide an opportunity to have a retrospective analysis of real-world data on the impact of RSV vaccine in preventing hospitalisation and death in elderly populations of all ages.

The efficacy of the RSV vaccine in subjects with a compromised immune system is still largely unknown and can also be informed by the retrospective data analysis from real-world data following the first phase of immunisation in England. The vaccine response usually decreases in very elderly subjects because of a weakened immune system due to aging and frailty or due to comorbidities or concomitant use of immunosuppressants inhibiting the immune system’s response to the vaccine. Further evidence may suggest if a seasonal vaccine (instead of the proposed all-year programme) or an additional dose may help boost vaccine efficacy in immunocompromised or those over 80s. Trials in immunocompromised individuals may also suggest if adjuvanted (Arexvy® by GSK) or mRNA (mRESVIA ® by Moderna)-based RSC vaccines were to provide superior protection in these individuals, compared to the unadjuvanted RSV vaccine (Abrysvo® by Pfizer). Only Pfizer’s vaccine has been procured and deployed in the phase-1 rollout of the RSV programme in England, this was a decision based on costs, as all three vaccines (Pfizer, GSK and Moderna) were deemed similar in efficacy by JCVI from the preliminary efficacy dataset available at the time.

Only licensed for use in newborns and children at the moment, Beyfortus® (nirsevimab) from AstraZeneca/Sanofi partnership contains monoclonal antibodies (mAbs) and provides passive immunity against RSV infection to neonates and children during their first RSV season. This can be another potential option for very elderly subjects or those with compromised immune systems if RSV vaccines from Pfizer, GSK or Moderna are not able to provide sufficient protection. Beyfortus (mAb) may also have a potential for use as a therapeutic for severely ill RSV patients by neutralising the circulating virus (reducing viral load); however, this is subject to clinical trials, as its efficacy in treating RSV remains largely unknown at this time.
